# Phonons take control of heat transfer in a metal

**DOI:** 10.1093/nsr/nwag190

**Published:** 2026-03-26

**Authors:** David G Cahill

**Affiliations:** Department of Materials Science and Engineering and Materials Research Laboratory, University of Illinois Urbana-Champaign, USA

Thermal and electrical conductivity are among the most fundamental physical properties of matter and have served as a testing ground for materials science since the nineteenth century. High conductivities—large heat and charge currents driven by temperature gradients and electric fields—are essential for applications in power transmission, thermal management and energy conversion. The best electrical conductors, such as Cu and Ag, are also excellent thermal conductors because charge and energy transport in metals are both carried by electronic excitations. Phonons, the wave-like lattice vibrations of crystals, also contribute to heat transfer; the phonon contribution to thermal conductivity is small in conventional metals and large in non-metallic crystals with strong covalent bonds, e.g. diamond and SiC.

In a recent publication [1], Sun and co-workers report a remarkable observation: a hexagonal phase of tantalum nitride, θ-TaN, bridges the gap between nonmetallic materials with high phonon thermal conductivity and conventional metals with dominant electronic heat transport. Their experiments show that θ-TaN combines metallic electrical conductivity with an exceptionally high phonon thermal conductivity of 500 W/(m K) at room temperature. They further argue that this value is likely not intrinsic and could be enhanced by suppressing nitrogen vacancy defects.

Although rigorous mathematical descriptions of electrical and thermal conductivity have existed for decades, quantitative predictions became feasible only with the development of first-principles computational methods and numerical solutions to transport equations. These tools have enabled theorists to identify extremes in the properties of materials that are rapidly tested experimentally. A recent success story is the compound semiconductor BAs [2]. More recently, advances in theory have allowed accurate treatment of higher-order phonon scattering processes and electron–phonon interactions. In 2021, Li and co-workers predicted that θ-TaN should exhibit a phonon-dominated thermal conductivity approaching 1000 W/(m K), while retaining significant electrical conductivity [3]. This unusual behavior arises from an anomalously low electronic density of states at the Fermi level, which weakens electron–phonon coupling, enabling both long phonon lifetimes and high electron mobility.

The primary challenge for experimental realization is the synthesis of the metastable θ phase of TaN with high purity and minimal crystalline defects. Sun and co-workers [1] employed high-temperature, high-pressure synthesis methods similar to those used commercially to produce diamond, generating sufficient material for neutron diffraction and electrical transport measurements on polycrystalline sintered pellets. A complementary approach was recently reported by Hu and co-workers [4], who grew θ-TaN crystals from a Na metal flux. Their measured thermal conductivity closely approaches the theoretical prediction, indicating exceptionally low defect densities. In both cases, however, the available θ-TaN crystals remain small, with lateral dimensions below 100 microns.

The work of Sun *et al.* represents a significant advance in the search for materials that defy conventional expectations. The unusual materials physics of θ-TaN is likely to support a rich spectrum of future studies. Beyond establishing its intrinsic transport coefficients, the coexistence of high-mobility metallic electrons and long-lived phonons suggests that θ-TaN may also exhibit unexpected thermoelectric behavior [5].

## References

[1] Liu Y, Zhou X, Pang G et al. Natl Sci Rev 2026; 13**:** nwag106. 10.1093/nsr/nwag106 42078949 PMC13131223

[2] Hou S, Pan F, Shi X et al. Phys Rev B 2025; 111: 235203. 10.1103/PhysRevB.111.235203

[3] Kundu A, Yang X, Ma J et al. Phys Rev Lett 2021; 126: 115901. 10.1103/PhysRevLett.126.115901 33798386

[4] Li S, Su C, Qin Z et al. Science 2026; 391: 707–11. 10.1126/science.aeb1142 41538411 PMC13371953

[5] Li C, Broido D. Mater Today Phys 2025; 53: 101706. 10.1016/j.mtphys.2025.101706

